# An organic acid-tolerant *HAA1*-overexpression mutant of an industrial bioethanol strain of *Saccharomyces cerevisiae* and its application to the production of bioethanol from sugarcane molasses

**DOI:** 10.1186/2191-0855-3-74

**Published:** 2013-12-30

**Authors:** Takuya Inaba, Daisuke Watanabe, Yoko Yoshiyama, Koichi Tanaka, Jun Ogawa, Hiroshi Takagi, Hitoshi Shimoi, Jun Shima

**Affiliations:** 1 Research Division of Microbial Sciences, Kyoto University, Kitashirakawa-oiwakecho, Sakyo-ku, Kyoto 606-8502, Japan; 2 National Research Institute of Brewing, 3-7-1 Kagamiyama, Higashihiroshima, Hiroshima 739-0046, Japan; 3 Division of Applied Life Sciences, Graduate School of Agriculture, Kyoto University, Kitashirakawa Oiwake-Cho, Sakyo-ku, Kyoto 606-8502, Japan; 4 Graduate School of Biological Sciences, Nara Institute of Science and Technology, 8916-5 Takayama, Ikoma, Nara 630-0192, Japan

**Keywords:** *Saccharomyces cerevisiae*, Ethanol fermentation, Sugarcane molasses, Acetate tolerance, *HAA1* gene

## Abstract

Bacterial contamination is known as a major cause of the reduction in ethanol yield during bioethanol production by *Saccharomyces cerevisiae*. Acetate is an effective agent for the prevention of bacterial contamination, but it negatively affects the fermentation ability of *S. cerevisiae*. We have proposed that the combined use of organic acids including acetate and lactate and yeast strains tolerant to organic acids may be effective for the elimination of principally lactic acid bacterial (LAB) contamination. In a previous study employing laboratory *S. cerevisiae* strains, we showed that overexpression of the *HAA1* gene, which encodes a transcriptional activator, could be a useful molecular breeding method for acetate-tolerant yeast strains. In the present study, we constructed a *HAA1*-overexpressing diploid strain (*MAT***a**/α, named ER HAA1-OP) derived from the industrial bioethanol strain Ethanol Red (ER). ER HAA1-OP showed tolerance not only to acetate but also to lactate, and this tolerance was dependent on the increased expression of *HAA1* gene. The ethanol production ability of ER HAA1-OP was almost equivalent to that of the parent strain during the bioethanol production process from sugarcane molasses in the absence of acetate. The addition of acetate at 0.5% (w/v, pH 4.5) inhibited the fermentation ability of the parent strain, but such an inhibition was not observed in the ethanol production process using ER HAA1-OP.

## Introduction

The utilization of bioethanol as an alternative to fossil fuels has attracted much attention in the effort to combat global warming and improve energy reserves. At present, bioethanol production generally utilizes derivatives from food crops such as sugar cane and corn grain (Cardona and Sanchez 2007). Bacterial contamination is known as a major cause of reductions in the yield of ethanol from such feedstock by yeast, *Saccharomyces cerevisiae* (Skinner and Leathers 2004). Briefly, these bacterial contaminants grow under conditions suitable for the growth of yeast, and reduce ethanol yields by competing for sugars such as glucose (Beckner et al. 2011). It has also been reported that the competition for nutrients between yeast and bacteria is a major reason for the loss of ethanol and viability in contaminated industrial ethanol production (Bayrock and Ingledew 2004).

Various agents have been examined for their potential to control bacteria and thereby avoid the reduction of ethanol yields. It has been reported that potassium metabisulfite and antibiotics effectively inhibit bacterial contamination (Aquarone 1960; Bayrock et al. 2003; Hynes et al. 1997; Oliva-Neto and Yokoya 1998; William and Carl 1986). In fact, antibiotics such as penicillin and virginiamycin are used in commercial bioethanol production today (Bayrock et al. 2003; Hynes et al. 1997). However, the addition of antibiotics to the broth may not be preferable from an ecological viewpoint, because the waste generated during bioethanol production should be recycled as useful products including forage or fertilizer. Antibiotics in the waste can lead to the emergence and spread of mutants resistant to antibiotics, which would threaten the safety of food and human health. Therefore, it is important to develop a method to control contamination by bacteria during bioethanol production without the use of antibiotics. In designing a bioethanol production process that eliminates bacterial contamination, we focused on acetate as a control agent.

Among bacteria that contaminate sugarcane juice, lactic acid bacteria (LAB) may be the most serious because of their rapid growth (Thomas et al. 2001). It is reported that particular *Lactobacillus* strains such as *Lb. plantarum* are the most abundant isolates from various commercial plants used in ethanol production (Saithong et al. 2009). Lactate produced by contaminating LAB has been reported as a strong inhibitor of ethanol production by yeast strains (Beckner et al. 2011). It can thus be considered that lactate tolerance is a critical characteristic for bioethanol yeast.

At elevated levels, acetate strongly inhibits bacterial growth and viability; however, the addition of high concentrations of acetate may also reduce the fermentation ability of *S. cerevisiae*. At an external pH below the pKa value (Lambert and Stratford 1999), the acetate predominantly assumes an undissociated lipophilic form and can permeate the plasma membrane by simple diffusion. At natural cytosolic pH, dissociation of the acids leads to the release of protons and the respective anions, which induces intracellular acidification (Kawahata et al. 2006). To overcome these conflicting effects of acetate addition, we attempted to construct acetate-tolerant yeast strains whose fermentation abilities were not affected by acetate. Previously, we proposed that a fermentation system using the acetate-tolerant yeast *Schizosaccharomyces pombe* under an acetate-containing condition was effective for preventing bacterial contamination (Saithong et al. 2009). However, the fermentation ability of *Sc. pombe* is much lower than that of *S. cerevisiae*. In this study, we attempted to construct acetate-tolerant strains from the industrial *S. cerevisiae* strain Ethanol Red (ER).

In previous reports, we described a breeding strategy for the improvement of acetate tolerance (Haitani et al. 2012; Tanaka et al. 2012). We determined by DNA microarray analysis that overexpression of *HAA1* gene, which is a transcriptional activator involved in the adaptation to weak acid stress, was up-regulated in the presence of acetate. It is known that Haa1 binds to an acetic acid-responsive element (ACRE), activating the expression of several targets, including the membrane transporter genes *TPO2* and *TPO3* (Fernandes et al. 2005; Keller et al. 2001; Mira et al. 2010). We have reported that a laboratory strain of *S. cerevisiae* that overexpressed the *HAA1* gene acquired a higher level of acetate tolerance (Tanaka et al. 2012).

In this study, we constructed an acetate-tolerant strain derived from the bioethanol yeast *S. cerevisiae* ER. Because acetate shows a negative effect on the growth of contaminated bacteria (Saithong et al. 2009) and is a safe and inexpensive reagent for inhibiting bacterial growth, we examined whether the combined use of acetate and a strain tolerant to acetate would be suitable for industrial ethanol production.

## Materials and methods

### Media

YPD medium (2% glucose, 1% yeast extract [Difco Laboratories, Detroit, MI, USA], and 2% peptone [Difco]) was used for yeast culture, and SD medium (2% glucose and 0.67% yeast nitrogen base without amino acids [Difco]) was used to isolate transformants. YPD medium (2% glucose, 1% yeast extract [Difco], and 2% peptone [Difco]) was used for determination of the fermentation characteristics of the constructed strain.

Sugarcane molasses was purchased from Matsuhisa (Gifu, Japan). Molasses medium (16% total sugar, calculated as the sum of sucrose, glucose and fructose, plus 0.046% KH_2_PO_4_ and 0.225% urea) was used for fermentation tests.

### Oligonucleotide primers

The sequences of all oligonucleotide primers used for PCR in this study are listed in Additional file 1: Table S1.

### Construction of the *HAA1*-overexpressing strain

As the parent strain, we employed ER (kindly provided by Le Saffre), which has been developed for industrial ethanol production. Two haploid strains derived from ER, ER-6c (*MAT***a**) and ER-3a (*MAT* α), and their *ura3*Δ*0* derivatives were used to construct the *HAA1*-overexpressing strain (Sasano et al. 2012). The genotypes of the yeast strains used in this study are summarized in Table 1.

**Table 1:** Yeast strains used in this study

**Strain**	**Description**	**Source or reference**
ER (Ethanol Red)	*MAT ***a**/α (wild type)	Le Saffre, France
ER-6c	*MAT ***a** derived from ER	(Sasano et al. 2012)
ER-6c ura3 Δ*0*	*MAT ***a***ura3Δ0* derived from ER-6c	(Sasano et al. 2012)
ER-3a	*MAT* α derived from ER	(Sasano et al. 2012)
ER-3a ura3 Δ*0*	*MAT* α *ura3Δ0* derived from ER-3a	(Sasano et al. 2012)
ER-6c HAA1-OP	*MAT ***a** P_*TDH3*_*-HAA1* derived from ER-6c ura3 Δ*0*	This study
ER-3a HAA1-OP	*MAT* α P_*TDH3*_*-HAA1* derived from ER-3a ura3 Δ*0*	This study
ER HAA1-OP	*MAT ***a/**α P_*TDH3*_*-HAA1*/P_*TDH3*_*-HAA1* constructed by mating between ER-3a HAA1-OP and ER-6c HAA1-OP	This study

*HAA1*-overexpressing strains were constructed on the basis of a previously reported method (Hasegawa et al. 2012) using the primer set listed in Additional file 1: Table S1 in the supplemental material. Briefly, the promoter region of the *TDH3* gene, which allows constitutive expression at a high level, was fused with the *URA3* marker gene and then inserted upstream of the start codon of the *HAA1* gene. Fusion PCR was performed according to the method previously described (Kuwayama et al. 2002). Yeast strains overexpressing the target genes were constructed by replacing the promoter region of each gene with that of *TDH3* gene, which encodes glyceraldehyde-3-phosphate dehydrogenase (GPDH).

These fragments were transferred into ER-6c *ura3*Δ*0* and ER-3a *ura3*Δ*0*. The fragments contained consensus sequences for each target gene at both ends; therefore, they were inserted into the chromosomal DNA of the host cells through homologous recombination. The intended homologous recombination was confirmed by PCR reactions using a set of PCR primers (HAA1 check-F and HAA1 check-R; shown in Additional file 1: Table S1).

Diploid strains were constructed by mating haploid strains of opposite mating types in YPD medium. Overnight cultures of each haploid parent were mixed and incubated at 30°C for 4 h without shaking. The mixtures were diluted 50-fold with fresh YPD medium. After cultivation overnight at 30°C, the mating mixture was plated onto YPD agar. Diploid strains were selected on the basis of colony size (Shima et al. 1999), yielding strain ER HAA1-OP. Diploid formation was confirmed based on spore formation ability and PCR analysis of the *MAT* loci (Katou et al. 2008).

### Quantitative real-time PCR analysis

To determine the expression level of the *HAA1* gene in each overexpressing strain, quantitative real-time PCR (qRT-PCR) analysis was performed. Total RNAs were extracted from logarithmically growing cells using the hot phenol method. Synthesis of the cDNAs from the total RNAs was performed using a PrimeScript II High Fidelity RT-PCR kit (Takara, Ohtu, Japan) with an oligo dT primer. Real-time PCR was carried out using a FastStart Essential DNA Green Master (Roche, Mannheim, Germany) and LightCycler nano (Roche). Data analysis was performed using the LightCycler Nano software version 1.0 (Roche). The primers used for the real-time PCR analysis are listed in Additional file 1: Table S1. The *TAF10* gene was employed as a control housekeeping gene, because *TAF10* gene is reported to be among the most suitable genes for use as a reference in qRT-PCR (Teste et al. 2009).

### Organic acids stress tolerance test

To test the acetate and lactate tolerance of the yeast transformants, each yeast strain was cultured in YPD medium until OD_600_ ~4-5 and then serially diluted, 10-fold at a time, and 4 μl of each diluted solution was applied to YPD agar medium containing 0.5% (w/v) acetate (pH 4.5) or 9% (w/v) lactate (pH 2.8). The samples were incubated at 30°C.

### Measurement of sugar, ethanol and acetate in the culture medium and molasses

Ethanol, glucose and acetate concentrations were determined using an HPLC system (Shimadzu, Kyoto, Japan) equipped with a fermentation monitoring column (Bio-Rad Laboratories, Hercules, CA, USA) and Micro-Guard Cation H Refill Cartridges with a Standard Cartridge Holder (Bio-Rad Laboratories), and detected using an RID-10A refractive index detector (Shimadzu). The column was kept at 60°C using a CTO-20A column oven (Shimadzu). Sulfuric acid solution (5 mM) was used as the mobile phase at a constant flow rate of 0.6 mL/min. Portions (10 μL) were injected into the HPLC system with an SIL-20A autosampler (Shimadzu), and each run was stopped at 13.4 min after the injection. The concentrations of the sugars and ethanol were determined using a standard curve generated by a series of external standards.

### Fermentation characteristics of the yeast strains in YPD medium

Strains ER and ER HAA1-OP were inoculated in YPD medium (100 ml in a 300-ml Erlenmeyer flask) containing 0% or 0.5% acetate (pH 4.5). The initial densities of the yeast cells were 4.8 × 10^7^ (equivalent to OD_600_ = 1). The cultures were incubated at 30°C for 18 h with shaking at 80 rpm, and the changes in ethanol, glucose and acetate in the culture medium during cultivation were monitored as described above.

### Ethanol production from sugarcane molasses under the acetate-addition condition

Strains ER and ER HAA1-OP were inoculated in molasses medium (100 ml in a 300-ml Erlenmeyer flask) containing 0% or 0.5% acetate (pH 4.5). The initial densities of the yeast cells were 4.8 × 10^7^ (equivalent to OD_600_ = 1). The cultures were incubated at 30°C for 24 h with shaking at 80 rpm, and the amounts of ethanol in the culture medium during cultivation were monitored as described above.

## Results

### Construction of a diploid *HAA1*-overexpressing strain and confirmation of the *HAA1* gene expression level

**Figure 1:**
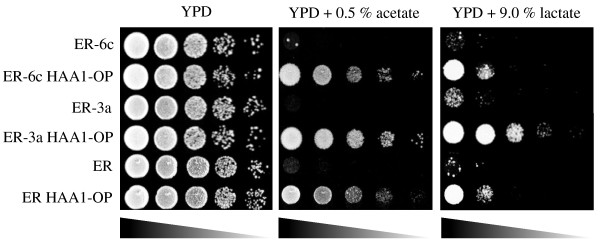
**Organic stress tolerance of *HAA1*-overexpressing strains.** The haploid strains (ER-6c, ER-6c HAA1-OP, ER-3a, ER-3a HAA1-OP) and diploid strains (ER and ER HAA1-OP) were spotted onto YPD medium, YPD medium containing 0.5% acetate (pH 4.5) and YPD medium containing 9.0% lactate (pH 2.6), and incubated at 30°C for 1 day, 2 days, and 6 days, respectively.

To construct a diploid strain overexpressing *HAA1* gene derived from *S. cerevisiae* ER, we employed a strategy of mating haploid strains that overexpressed the *HAA1* gene. Several haploid strains were obtained by tetrad dissections from ER and their fermentation patterns were analyzed. Because the fermentation abilities of ER-6c and ER-3a were intermediate between those of the other haploid strains, we used ER-6c and ER-3a as the *MAT***a** haploid strain and *MAT* α haploid strain, respectively (Sasano et al. 2012). To enable gene introduction in the haploid strains, *URA3* genes were deleted. In each uracil auxotrophic haploid strain, the promoter region of the native *HAA1* gene was replaced by a *TDH3* promoter, which is a constitutive strong promoter, resulting in ER-6c HAA1-OP and ER-3a HAA1-OP. To estimate the expression levels of *HAA1* gene in ER-6c HAA1-OP and ER-3a HAA1-OP, qRT-PCR analysis was carried out. The results indicated that the expression levels of these overexpressing strains were approximately 7–8 times higher than that of parent strain (Figure 1).

Mating was performed between strains ER-6c HAA1-OP and ER-3a HAA1-OP to obtain the diploid *HAA1*-overexpressing strain ER HAA1-OP (*MAT***a**/α). Diploid formation was confirmed by both spore formation ability and PCR analysis of *MAT* loci (data not shown). In Figure 1, the expression levels of the *HAA1* gene in ER HAA1-OP and parental cells grown in YPD medium are shown. These results show that the expression level of the *HAA1* gene in ER HAA1-OP is 6 times higher than that in the parent strain.

### Stress tolerance to acetate and lactate of the *HAA1*-overexpressing strain

**Figure 2:**
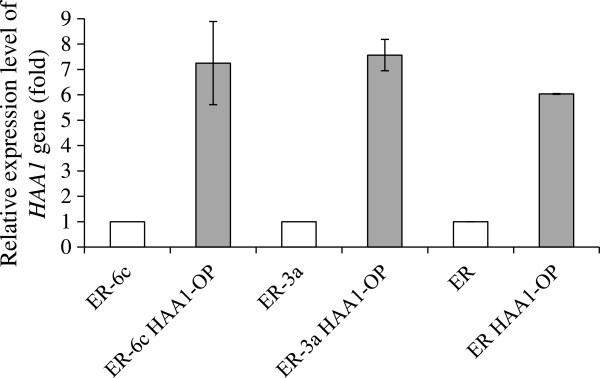
**Expression levels of *HAA1*-overexpressing strains.** Expression levels were monitored in RNAs extracted from cells of *S. cerevisiae* ER-6c, ER-6c HAA1-OP, ER-3a, ER-3a HAA1-OP, ER and ER HAA1-OP. The data presented in this figure are averages from three independent experiments. Error bars represent standard deviations.

To determine the effects of the overexpression of *HAA1* gene on stress tolerance to organic acids, the stress tolerance to acetate of ER HAA1-OP (Figure 2, middle panel) was evaluated. As expected, the *HAA1*-overexpressing haploid strains (ER-6c HAA1-OP and ER-3a HAA1-OP) and the diploid strain (ER HAA1-OP) showed drastically higher acetate tolerance than each parent strain.

We also found that the *HAA1*-overexpressing haploid strains (ER-6c HAA1-OP and ER-3a HAA1-OP) and the diploid strain (ER HAA1-OP) showed tolerance to lactate (Figure 2, right panel), although in our previous study employing a laboratory yeast strain, we did not observe lactate tolerance (Tanaka et al. 2012).

### Fermentation characteristics of strain ER HAA1-OP

**Figure 3:**
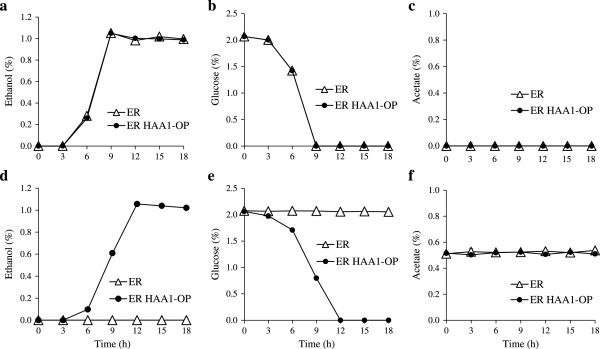
**Ethanol production, glucose consumption and acetate concentration of *S. cerevisiae *ER and ER HAA1-OP in YPD medium (a, b and c), and YPD medium containing 0.5% acetate (d, e and f).** Strains ER and ER HAA1-OP were inoculated in YPD medium (100 ml in a 300-ml Erlenmeyer flask) containing 0% or 0.5% acetate (pH 4.5). The initial densities of the yeast cells were 4.8 × 10^7^ (equivalent to OD_600_ = 1). The cultures were incubated at 30°C for 18 h with shaking at 80 rpm. The data presented in this figure are averages from three independent experiments. Error bars represent standard deviations.

To determine the fermentation characteristics of the strain ER HAA1-OP, changes in ethanol production, glucose consumption and acetate concentration in YPD with or without acetate addition at the concentration of 0.5% (pH 4.5) were examined (Figure 3). The pH values of the media were adjusted to 4.5, which is slightly below the pKa value (the pKa value of acetate is 4.76). Under the condition without acetate addition, such parameters of ER HAA1-OP were almost the same as those of the parent strain (Figure 3a, b and c). These data suggested that overexpression of the *HAA1* gene did not negatively impact the glucose fermentation ability. When the parent strain was used, acetate addition significantly inhibited ethanol production. However, the cultivation time when the maximum ethanol concentration of strain ER HAA1-OP was reached under the acetate-addition condition was 3 h longer than that under the non-stress condition (Figure 3a and d). The maximum ethanol concentrations (approximately 1.0%) were nearly the same in both ER and ER HAA1-OP.

### Ethanol production from sugarcane molasses by ER HAA1-OP

**Figure 4:**
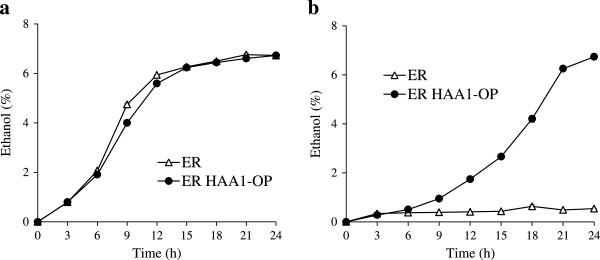
**Ethanol production of *S. cerevisiae *ER and ER HAA1-OP in molasses medium (a) and molasses medium containing 0.5% acetate (b).** Strains ER and ER HAA1-OP were inoculated in molasses medium (100 ml in a 300-ml Erlenmeyer flask) containing 0% or 0.5% acetate (pH 4.5). The initial densities of the yeast cells were 4.8 × 10^7^ (equivalent to OD_600_ = 1). The cultures were incubated at 30°C for 24 h with shaking at 80 rpm. The data presented in this figure are averages from three independent experiments. Error bars represent standard deviations.

To evaluate fermentation ability, ethanol production was monitored in sugarcane molasses, a useful feedstock (Figure 4). The sugarcane molasses used in these experiments did not contain significant amounts of either acetate (less than 0.1% of the total amount of molasses) or lactate (less than 1% of the total amount of molasses) (data not shown). We carried out ethanol production experiments in molasses medium containing 16% total sugar.

Figure 4a shows the ethanol production by ER HAA1-OP and its parent strain in molasses medium. The amounts of ethanol in the medium reached approximately 7% within 24 h of incubation using either ER HAA1-OP or its parent strain. These results suggested that ER HAA1-OP is suitable for ethanol production from sugarcane molasses. Figure 4b shows the ethanol production by ER HAA1-OP and its parent strain in molasses medium containing 0.5% acetate (pH 4.5, which is near the pKa value of acetate). The ethanol productivity of ER was significantly inhibited in the presence of 0.5% acetate. The ethanol production rate of ER HAA1-OP under the acetate-addition condition was lower than that under the non-stress condition. This was particularly true in the initial stage of cultivation (0–9 h after inoculation), when ethanol production was significantly inhibited (Figure 4b). Although the time required for the maximum ethanol concentration was different, the maximum ethanol concentration (approximately 7%) was nearly the same under both the acetate-addition condition and non-stress condition.

## Discussion

In this paper, we showed the possibility of designing an effective bioethanol production system with the addition of acetate. Previously, we reported that an *HAA1*-overexpressing strain derived from a laboratory strain showed higher acetate tolerance, and that the overexpression of *HAA1* gene induced the expression of Haa1-regulated genes, including *TPO2* and *TPO3*, the products of which are considered major facilitator superfamily transporters of the *S. cerevisiae* plasma membrane (Tanaka et al. 2012). In the present study, the constitutive *HAA1*-overexpressing diploid strain (ER HAA1-OP) was constructed from the industrial yeast strain *S. cerevisiae* ER. The ER HAA1-OP strain showed not only higher acetate tolerance by spot assay but also higher fermentation ability (ethanol production) in the presence of 0.5% acetate than the wild-type strain.

In our previous study, the same promoter was used in overexpressing the strain derived from the laboratory strain S288C (Tanaka et al. 2012). In the laboratory strain, the replacement of the native promoter of the *HAA1* gene by the *TDH3* promoter conferred an approximately 3-fold increase of *HAA1* gene expression. The effects of promoter replacement on the enhancement of *HAA1* expression were much greater under the genetic background of *S. cerevisiae* ER than that of S288C. We speculate that differences in the expression levels of *HAA1* between the laboratory strain (an approximately 3-fold increase) and *S. cerevisiae* ER (an approximately 6-fold increase) may have led to the different results in lactate tolerance. Because lactate produced by contaminated LAB is one of the possible inhibitory factors of ethanol production by yeast strains, this characteristic may be useful to avoid stuck fermentation. It is possible that the high-level expression of the *HAA1* gene in ER HAA1-OP may contribute to lactate tolerance. At present, we are not able to determine the reasons for these differences; however, it is possible that the gene expression levels of *TDH3*, which encodes proteins involved in glycolysis, are higher in the strains from an ER background than in the laboratory strain used here. In general, the fermentation ability of industrial strains is much higher than those of laboratory strains.

We consider the combined use of strains tolerant to organic acids, including acetate and lactate, to be suitable for industrial ethanol production from molasses, because organic acids are safe and inexpensive reagents for inhibiting bacterial growth. By making antibiotics unnecessary, our proposed ethanol production system offers several advantages. One of the most important is that the waste generated during antibiotic-free bioethanol production can be used safely as forage or fertilizer. In the future, inexpensive methods for preparing organic acids, such as recycling, need to be investigated for industrial application. However, ER HAA1-OP clearly has the ability to produce ethanol in molasses medium containing 0.5% acetate.

In future studies, it may be useful to pursue a bioprocess which eliminates the heat sterilization process of feedstock, which is one of the most energy-consuming steps in bioethanol production. We believe that one of the key technologies in the design of economic and environmentally friendly processes is the improvement of industrial microorganisms, such as yeast and bacteria, by enhancing characteristics such as their tolerance to environmental stress. To enable the breeding of such microorganisms, detailed elucidation of their stress-tolerance mechanisms will be needed.

## Competing interests

The authors declare that they have no competing interests.

## Authors’ contributions

JS designed research and wrote the manuscript. DW, HS and KT designed strains. IT and YY performed strain construction and fermentation assay. JO and HT commented and supervised on the manuscript. All authors read and approved the final manuscript.

## Supplementary Material

Additional file 1: Table S1 The sequence of oligonucleotide primers used in this study. Click here for file
