# Supplementary material for: An organic acid-tolerant HAA1-overexpression mutant of an industrial bioethanol strain of Saccharomyces cerevisiae and its application to the production of bioethanol from sugarcane molasses
Source: AMB Express. 2013 Dec 30;3:74. doi: 10.1186/2191-0855-3-74 (PMC3896689; doi:10.1186/2191-0855-3-74)
Supplement: Additional file 1: Table S1 — The sequence of oligonucleotide primers used in this study. [file 2191-0855-3-74-S1.doc]

Electronic supplementary file

AMB express

**An organic acid-tolerant *HAA1*-overexpression mutant of an industrial bioethanol strain of *Saccharomyces cerevisiae* and its application to the production of bioethanol from sugarcane molasses**

Takuya Inaba^1, 3^, Daisuke Watanabe^2,4^, Yoko Yoshiyama^1^, Koichi Tanaka^1^, Jun Ogawa^3^, Hiroshi Takagi^4^, Hitoshi Shimoi^2^, , Jun Shima^1^*

^1^ Research Division of Microbial Sciences, Kyoto University, Kitashirakawa-oiwakecho, Sakyo-ku, Kyoto 606-8502, Japan

^2^ National Research Institute of Brewing, 3-7-1 Kagamiyama, Higashihiroshima, Hiroshima 739-0046, Japan

^3^ Division of Applied Life Sciences, Graduate School of Agriculture, Kyoto University, Kitashirakawa Oiwake-Cho, Sakyo-ku, Kyoto 606-8502, Japan

^4^ Graduate School of Biological Sciences, Nara Institute of Science and Technology, 8916-5 Takayama, Ikoma, Nara 630-0192, Japan

*Corresponding author: Research Division of Microbial Sciences, Kyoto University, Kitashirakawa-oiwakecho, Sakyo-ku, Kyoto 606-8502, Japan, Phone: +81-75-753-9545-8016; Fax: +81-75-753-9544; E-mail: shimaj@kais.kyoto-u.ac.jp

Table S1. The sequence of oligonucleotide primers used in this study.

| Primer | Sequence(5’→3’) |
| --- | --- |
| For construction of the *HAA1*-overexpressing strain | |
| URA3-F1 | CAGGGTCCATAAAGCTTT |
| URA3-R1 | TTTATAAAGGCCATGAAGCT |
| TDH3 pro-F | GGAAAGAAAAAGCTTCATGGCCTTTAT AAAAACACGCTTTTTCAGTTC |
| TDH3 pro-R | TTTGTTTGTTTATGTGTGTT |
| HAA1-A | GCTTCCGGTAATTTGCGA |
| HAA1-B | AAGATGAATTGAAAAGCTTTATGGACC CTGCCTTTTTCTTGTCAAATATGGTAG |
| HAA1-C | GTTTCGAATAAACACACATAAACAAAC AAAATGGTCTTGATAAATGGCATAAAG |
| HAA1-D | CTTGCCAGTTATTGCCTG |
| For confirmation of intended homologous recombination | |
| HAA1 check-F | GGTTCGATTCCGGGCTTG |
| HAA1 check-R | CTTGCCAGTTATTGCCTG |
| For confirmation of mating type by PCR analysis | |
| MAT locus-F | AGTCACATCAAGATCGTTTATGG |
| MAT a-R | ACTCCACTTCAAGTAAGAGTTTG |
| MAT α-R | GCACGGAATATGGGACTACTTCG |
| For detection of the expression level of *HAA1* gene by qRT-PCR analysis | |
| TAF10-RTPCR-FW | CCAGGATCAGGTCTTCCGTA |
| TAF10-RTPCR-RV | CAACAGCGCTACTGAGATCG |
| HAA1-RTPCR-FW | GACATCGGAAAGGGAGTTGA |
| HAA1-RTPCR-RV | GGAGATGTCGCTACGGTTGT |
